# Lamellar Orientation Analysis and Mechanical Properties of Polyethylene in Stretch-Induced Crystallization

**DOI:** 10.3390/polym17111450

**Published:** 2025-05-23

**Authors:** Mohammed Althaf Hussain, Takeshi Aoyagi, Takeshi Kikutani, Wataru Takarada, Takashi Yamamoto, Syed Farooq Adil, Shigeru Yao

**Affiliations:** 1 Faculty of Engineering, Fukuoka University, Fukuoka 814-0180, Japan; 2 Informatics Initiative (IFX), Asahi Kasei Corporation, Chiyoda 100-0006, Japan; aoyagi.tc@om.asahi-kasei.co.jp; 3 Institute of New Industry Incubation, Institute of Science Tokyo, Yokohama 226-8503, Japan; kikutani.t.aa@m.titech.ac.jp; 4 Faculty of Textile Science and Technology, Shinshu University, Ueda City 386-8567, Japan; 5 Graduate School of Science and Engineering, Yamaguchi University, Yamaguchi 753-8512, Japan; 6 Department of Chemistry, College of Science, King Saud University, P.O. Box 2455, Riyadh 11451, Saudi Arabia

**Keywords:** stretch-induced crystallization, morphology, lamellar orientation, entanglements, mechanical properties

## Abstract

Polyethylene films prepared from orientation-dependent methods are strong and resilient, have reduced permeability, and possess higher tensile strength. A molecular dynamics investigation is performed to reveal the emergence of chain folding and lamellar crystal axis alignment along the stretching axis (tilt angle) in the stretch-induced crystallization (SIC) of high-density polyethylene (HDPE), which mimics the internal structure of the fiber. The morphology in phase transition is assessed by the total density (*ρ*), degree of crystallinity (%$\chi_{c}$), average number of entanglements per chain (<*Z*>), elastic modulus of the mechanical property, and lamellar chain tilt angle (θ) from the stretch-axis. The simulation emphasizes crystal formation by changing the total *ρ* from 0.85 g·cm^−3^ to 0.90 g·cm^−3^ and by tracking the gradual increase in %${\;\chi }_{c}$ during stretching (~40%) and relaxation processes (~50%). Moreover, the primitive path analysis-based <*Z*> decreased during stretching and further in the subsequent relaxation process, supporting the alignment and thickening of the lamellar chain structure and chain folding from the random coil structure. The elastic modulus of ~350–400 MPa evidences the high alignment of the lamellar chains along the stretching axis. Consistent with the chain tilt angle of the HDPE in SAXS/WAXS experiments, the model estimated the lamellar chain title angle (*θ*) relative to the stretching axis to be ~20–35°. In conclusion, SIC is a convenient approach for simulating high stiffness, tensile strength, reduced permeability, and chain alignment in fiber film models, which can help design new fiber morphology-based polymers or composites.

## 1. Introduction

The crystallization behavior of semicrystalline polymers has been explored under various conditions, including temperature, strain, and flow [1,2,3,4,5,6,7,8]. The unique structural features of semicrystalline polymer chains are attributed to the distribution and direction of random amorphous and ordered lamellar conformations. Tensile deformation exhibits the viscoelastic characteristics of plastics, comprising both elastic and plastic regimes when tensile deformation forces are applied [9,10,11,12]. It has been known that a marginal variation in the composition of amorphous and lamellar portions can amend the morphology-dependent properties of polymers. A balance or specific ratio between elastic and plastic regimes is crucial for informing industrial product development [13,14]. Polymer films exhibiting superior ductility and improved plastic regimes in their mechanical properties should have a high proportion of amorphous content. Generally, a high-amorphous-phase product in semicrystalline polymers is flexible, transparent, and used in packaging applications [11,12,15,16,17,18,19]. Alternatively, the material could also be brittle, resembling high-performance polymer fibers, when the imbalanced proportion favors the crystalline proportions [11,12,16,17,18,19]. The high content of polymer chain alignment shows an elevated elastic modulus and resistance to tensile forces, which could be ascribed to the morphological differences dominated by the lamellar chain orientation and chain folding structure.

Although highly stretched polymer chain fiber materials, in which the lamellae are highly aligned, might have attractive applications, controlling alignment and orientation during crystallization remains challenging, despite the availability of state-of-the-art technical equipment. Fortunately, fiber spinning, film blowing, machine direction orientation (MDO), and film stretching process techniques in real-world applications have been widely used for lamellar-oriented morphology product developments [19,20,21,22,23,24]. The films processed by the above methods have promising mechanical properties, resilience, and reduced permeability [23,24]. Thus, we aimed to mimic such polymer processing techniques using uniaxial molecular dynamics (MD) simulations to prepare the highly aligned lamellar stems in the desired direction [23,24,25]. This simulation study reveals the morphological evolution during stretching and relaxation to obtain a well-aligned polymer chain model with a balanced proportion of lamellar and amorphous regions. Moreover, the lamellar chain tilt angle (*θ*) aligning with the stretching axis has been evaluated to predict product characteristics during simulation processes involving stretching and relaxation.

Nevertheless, several MD simulations have been reported, discovering the lamellar orientation-dependent morphology that results in the formation of fibril film models. Theodorou et al. [1,2] recently reported morphological changes in MDO processing techniques, but the processing approach is a planar extension. This study sheds light on crystal kinetics and morphology differences under a range of strain rates and temperatures greater than 325 K. Both parameters (strain rate and temperature) are crucial in polymer processing and crystallization-dependent applications. Using uniaxial elongational flow by the Monte Carlo method, Baig and Edwards developed a flow-induced crystalline (FIC) phase in isotropic polymeric liquids at 450 K, 400 K, 350 K, and 300 K [26]. The simulated FIC structure demonstrated real crystallite characteristics, which is an anticipated cornerstone of simulations. Furthermore, Yamamoto documented the fibril structure with crystal network formation in uniaxial deformation, which highlights the topological features of the semicrystalline polymer crystal network [4].

Various lamellae distribution and orientation analyses revealed a strong relationship between morphology, specifically inner-chain orientation, and mechanical properties. An experimental investigation by Kanomi et al. [27] reported that the chain tilt angle (*θ*) for isolated lamellae favored an angle of 34°, and *θ* was smaller for stacked lamellae. The authors claimed that the distinct physical and mechanical properties of stacked and isolated lamellar crystals are apparently due to their differing *θ* in the morphology [28,29]. Nevertheless, much deeper insights into *θ* have been revealed by MD simulations of semicrystalline HDPE materials [2,30,31]. *θ* has also been predicted in the SIC-HDPE model by Romanos et al. [2]. The lamellar *θ* was discovered to lie ca. 20–24° from the pulling direction or elongated direction, which is due to the relaxation of the model after removing the applied mechanical forces of the recrystallized models in the stretching process. Moreover, flow condition-dependent simulations by Nicholson and Rutledge proved the reduction in tilt angle during shear and uniaxial extensional flows, which led to long-chain alignment and enhanced nucleation. [30] Supporting this, Gautam and Rutledge have shown that modulation of chain tilt affects both stability and crystallinity [31]. Since thermal history and shear strain–thermal history polymer processing morphology play a critical role in morphology-dependent properties, a stretching and relaxing temperature of 370 K could be an optimal choice for observing crystallization patterns, with increased chain mobility in the HDPE chains and further providing dual insights for its practical applications. This contrasts with the crystallization temperatures recommended by usual simulations reported at 350 K and by several studies. [31] A few high-temperature (>350 K) stretching process studies by Baig et al. [26] at 450 K and 400 K and Theodorou et al. [1] at 365 K are available. Still, information on tilt angle and mechanical properties remains limited. Compiling the above information encouraged us to conduct the present study, highlighting the significant results of morphology and mechanical property changes with highly movable PE chain models, typically achieved by higher-temperature stretching and relaxation simulations at 370 K.

A monodisperse (HDPE) semicrystalline model with amorphous and highly ordered crystalline regions has been prepared using stretch-induced crystallization (SIC), followed by relaxation at 370 K from the isotropically coiled model at 450 K. Microstructure characterization is performed by evaluating the physical and mechanical properties. Previous molecular dynamics studies on polyethylene semi-crystallization have focused on quiescent crystallization at room temperature or rapid crystallization near melting temperatures. [11,12,32,33,34,35,36,37,38,39,40] In contrast, stretch-induced crystallization of semicrystalline HDPE at 370 K represents a critical yet underexplored area. This regime bridges melt-state mobility and ordering-driven crystallization, enabling the analysis of chain orientation, lamellar development, and relaxation processes under realistic conditions. Moreover, rapid crystallization is a crucial part of SIC that may occur in experimental fiber spinning experiments at this temperature regime, and it can be a guide for predicting pre-nucleus formation. Such insights link molecular dynamics to polymer processing pathways, positioning this study to advance understanding.

## 2. Simulation Methodology

### 2.1. Simulation Models

Monodisperse HDPE chains comprising 1000 united atoms (UAs) were packed into a cubic box with dimensions of a = b = c = 64.956 Å using Enhanced Monte Carlo (EMC) simulation code [41] at T = 300 K and P = 1 atm. The final 10-chain simulation model comprises 10,000 united atoms (10C_1000_) and has a molecular weight of 140,000 amu. Three initial independent models were considered in this study, and their averages are discussed. The average volume of the boxes is ~274,063.47 Å^3^. The randomly distributed PE model density was 0.85 g·cm^−3^ [11,12]. To maintain the entanglement density of the HDPE in the amorphous state of semicrystalline materials, similar to the experiments, a molecular weight of 14,000 amu was considered for each polymer chain. HDPE models were chemically represented as CH_2_ and CH_3_, where CH_2_ represents backbone chain atoms; on the other hand, CH_3_ refers to the terminal chain atoms whose molecular weights are 14.02 and 15.03 amu, respectively.

### 2.2. Computational Methods

The large atomic–molecular massively parallel simulation (LAMMPS) code in its default form was used for all calculations [42]. The interatomic forces among the HDPE polymer chains were represented using the Transferable Potential for Phase Equilibria (TraPPE) UA forcefield parameters, and the mathematical representation of the forcefield parameters is shown in the following equations [43,44,45,46,47]:

Bonded terms:

(1) $$E_{bond}=k_{b}(r-r_{0}{)}^{2}$$

(2) $$E_{angle}=k_{b}(\theta -\theta_{0}{)}^{2}$$

(3) $$E_{dihedral}=\sum_{0}^{n=3}C_{i}\;{(cos}^{i}\left(\phi \right))$$

Non-bonded terms:

(4) $$E_{Lennard-Jones}=4\varepsilon_{ij}\;\{(\sigma_{ij}/r_{ij}{)}^{12}-(\sigma_{ij}/r_{ij}{)}^{6}\}$$

where $\varepsilon_{ij}=\sqrt{\varepsilon_{i}\varepsilon_{j}};\sigma_{ij}=\sqrt{\sigma_{i}\sigma_{j}}$.

The bonded and non-bonded terms are computed as follows in Table 1.

These forcefield parameter units effectively illustrate molecular interactions and behavior: the force constant (K_b_) for bond potential is represented in kcal/mol/Å^2^; the force constant for angle potential (K_θ_) is given in kcal/mol/radian^2^; the dihedral potential (*C*_0_) is quantified in radians; the equilibrium angle (*θ*_eq_) is stated in degrees; the equilibrium bond length (*r*_eq_), range of the distance at which the LJ potential is zero (*σ*), and cut-off distance (rcut) are denoted in Å; the empirical parameters for the first harmonic term (*C*_1_), the amplitudes of the first (*C*_2_) and second cosine terms (*C*_3_), and the depth of potential energy (*ε*) are conveyed in kcal/mol.

The randomly packed cubic boxes were initially relaxed using the NVE ensemble for 100 ps using a 1 fs time step (Δt). In the following step, these models were heated using the canonical ensemble (NVT) to T = 450 K to prepare an isotropic coil structure by equilibrating it with 100 ns long relaxation using the NPT ensemble. Note that the NVT and NPT simulations were performed using Δt = 2 fs. To justify the steady-state formation of the model after 100 ns NPT equilibration at T = 450 K and P = 1 atm, the vdW energy, density, temperature, and pressure along the *Z*-axis were printed at an interval of 1 ns. LAMMPs’ default parameters were considered in all the calculations. To mimic the bulk state and remove the surface effect for the cubic model, three-dimensional periodic boundary conditions were applied in all simulations. The tail corrections accounted for long-range interactions to improve computational efficiency, and the mixing rules were used for Lennard–Jones parameters. As recommended by the TraPPE forcefield, a standard Lennard–Jones cut-off of 14 Å was used for non-bonded interactions. The pressure in all NPT simulations was 1 atm, except during mechanical property evaluations, which were conducted under zero pressure [11,12].

Once the isotropic structure was formed at 450 K, the models were slowly quenched to 400 K at a cooling rate of 10 K/1 ns. It further cooled slowly by 5 K/1 ns from 400 K to 370 K. After reaching 370 K through step-by-step temperature quenching methods, three models were again structurally relaxed at the same T (370 K) for another 10 ns to prepare the thermally uniformed model before stretching in the *Z*-axis direction. In the following step, the models were stretched to 300% of their initial structure in 300 ps time length simulations, and 3 ps interval trajectories were recorded for analysis. For crystallization of the stretched model, an NPT-100 ns relaxation was performed at 370 K in the subsequent step, and the trajectories were printed again at 1 ns intervals.

### 2.3. Characterization of the Models and Predictions of Mechanical Properties

#### 2.3.1. Lamellar Ordering and Degree of Crystallinity ($\chi_{c}$)

The melt-to-crystalline-state phase transformation during stretching and relaxation was measured by computing $\chi_{c}$ using the local orientation parameter. For the second-order Legendre polynomial ($P_{2})$ calculations, the coarse-grained molecular dynamics program (COGNAC) in the open computational tool for advanced materials technology (OCTA and J-OCTA) [48] was applied using in-house-developed Python code, version 3.6. Similar to our previous methods [11,12], Herman’s orientation factor (Equation (5)) was employed to calculate the degree of crystalline order $P_{2}\left(r\right)$ immediately after measuring the mid-point of two adjacent CH_2_-CH_2_ UA bonds; this mid-point is the ith UA located at the position r_i_.

(5) $$P_{2}\left(r\right)=\langle 3{cos}^{2}\theta_{i,j}-1\rangle /2$$

The angle (*θ*_i,j_) is defined between chord vectors bi and bj within the same mesh cell of 8 Å located at the representative position r. The average is taken over all pairs of chord vectors within the mesh cell. The crystallinity ($\chi_{c}^{mesh}$) is estimated by dividing the number of mesh cells Nc that have local order parameters greater than a threshold cut-off of 0.4 by the total number of mesh cells in the system N_total_. The cut-off of 0.4 is selected based on the recommendation by previous publications [49].

(6) $$\chi_{c}^{mesh}=N_{c}(P_{2}>0.4)/N_{total}$$

Similar strategies have been observed for calculating the order parameters and $\chi_{c}\;$ in previous publications [4,10,11,12,50].

#### 2.3.2. Evaluating the Mechanical Properties 

In general, the semicrystalline structures developed from SIC are anisotropic, requiring tensile tests in the x, y, and z directions of the simulation box. The semicrystalline models are uniaxially deformed in the transverse direction at a strain rate ($\dot{\varepsilon }$) of 10^10^ s^−1^ and a temperature of 370 K under 0 atm pressure, reaching 500% deformation over 1 ns. Stress–strain curves (SSCs) are generated for all deformation directions by recording molecular dynamics (MD) simulation trajectories at 1 ps intervals, and the time step (∆t) is set to 1 fs. The average data and moving averages of pressure tensors in the x (Pxx), y (Pyy), and z (Pzz) directions are evaluated to gauge the anisotropic mechanical behavior.

The strain rate ($\dot{\varepsilon }$) is computed using Equation (7), which represents the change in uniaxial deformation strain (ε) over time, expressed as the formation strain (∆ε) divided by the change in time (∆t).

(7) $$\dot{\varepsilon }=(∆\varepsilon /∆t)$$

whereas

(8) $$\varepsilon =(\left(L-{\;L}_{0}\right)/L_{0})=(∆L/L_{0})$$

$L_{0}$ and *L* are the initial and final lengths of the box along the deformed axis of the simulation box, which is measured using the Henchy strain.

To characterize the models, the density (ρ), temperature (T), pressure (P), van der Waals (vdW) energies, and pressure (Pxx/Pyy/Pzz) along all directions were computed using the LAMMPS code. In addition to the % $\chi_{c}$, the radius of gyration (R_g_) and chain end-to-end distances (R_ee_) were measured using the OCTA tools version 8.4. [48] The *Z1+* code was utilized for calculating the average ennoblements per chain (<*Z*>) in the equilibrated, quenched, stretched, and relaxed models. [51,52] All structures were visualized using the Ovito tools [53], and the data representation in the graph was prepared using Microsoft Excel version 2020 [54].

## 3. Results and Discussions

### 3.1. Structure, Equilibration, and Generation of an Isotropic Structure

**Figure 1:**
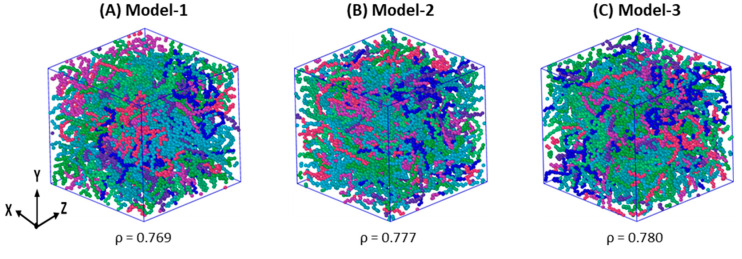
Representation of the equilibrated samples of the initial models at 450 K along with their densities (ρ in g·cm^−3^). All three models are equilibrated using the NPT ensembles at 450 K and 1 atm for a 100 ns time length. Each color represents one C_1000_-UA chain.

**Figure 2:**
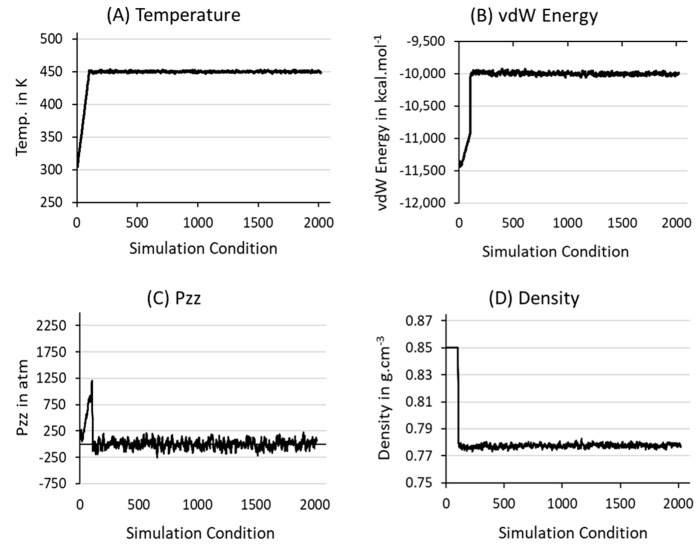
The simulation parameters represent the equilibration of the model at T = 450 K and P = 1 atm using an NPT ensemble for 100 ns. Before this, the model was melted using the NVT ensemble at the same T for 1 ns. All values are shown in the averages of the three-sample data.

Figure 1 emphasizes the NPT-100 ns simulated equilibrated structure of three independent HDPE models at 450 K and 1 atm, and their final ρ values are shown in g·cm^−^^3^. The distinct ρ data demonstrate the formation of three independent, well-equilibrated models which can produce unique SIC models upon stretching at a molding temperature of 370 K. The steady-state formation in 100 ns equilibration is quantitatively estimated as the average of the three models in Figure 2. The crucial polymer dynamic parameters reported here are temperature (T), van der Waals (vdW) energy, pressure tensors (Pzz) along the stretching Z-axis direction, and the total density (ρ) of the model. All the parameters in Figure 2 are relaxed to the desired value after an initial equilibration phase within the NPT-10 ns MD simulations, and any significant changes are unnoticed upon extension of the MD simulations up to a time length of 100 ns. The stabilization of the four independent terms indicates that the formation of the Gaussian chain coil structure, which meets the (R_ee,avg_)^2^)⁄(R(_g,avg_)^2^) ratio of 5.658, is close to the standard value of 6.0 for HDPE models [1,3]. Further, this ratio ensured that the production run of stretching and relaxation at 370 K can give reliable results, as evidenced by Ramanos and Salazaar et al. through comparisons between computational and experimental results [2,3].

Moreover, the density data support the formation of an isotropic structure after the 100 ns relaxation. From the simulation of the PE under the condition of T = 450 K and P = 1 atm, it is known that the typical ρ value of PE represented by the TraPPE-UA forcefield parameter is ~0.754 g·cm^−3^ [1,3,11,12,26]. From Figure 1, the average ρ value is 0.775 g·cm^−3^ at T = 450 K and P = 1 atm, indicating that the NPT-100 ns equilibration of HDPE models at 450 K stabilized as an isotropic melt structure. Furthermore, the average chain end-to-end distance (R_e-e_) is 136.3 Å, representing a coil structure formation, which mimics an isotropic melt or a well-equilibrated polymer structure, suitable for production (SIC) simulations [2,3].

### 3.2. Morphology

#### 3.2.1. Stretching and Relaxation of the Model at 370 K for Crystallization

The thermodynamic parameters of Figure 2 justified that the model is isotropically melted at 450 K. The SIC model is prepared as follows: First, the gradual quenching process is started from 450 K to 400 K with a cooling rate of 10 K/1 ns and thereafter (from 400 K) to 370 K with a cooling rate of 5 K/1 ns to relax the polymer chains slowly by gradually reducing the temperature. Then, the system is further relaxed at the same temperature for another 10 ns using NPT simulation to ensure the formation of the well-relaxed structure. Afterward, the investigation is extended to prepare a lamellar chain-aligned semicrystalline model at 370 K by induced forces like stretching, ensuring that the HDPE chains along the *Z*-axis are stretched up to 300% of the simulation box length from the initial point of stretching. Finally, NPT-100 ns relaxation simulations were performed to thicken the extended lamellar stems along the *Z*-axis.

**Figure 3:**
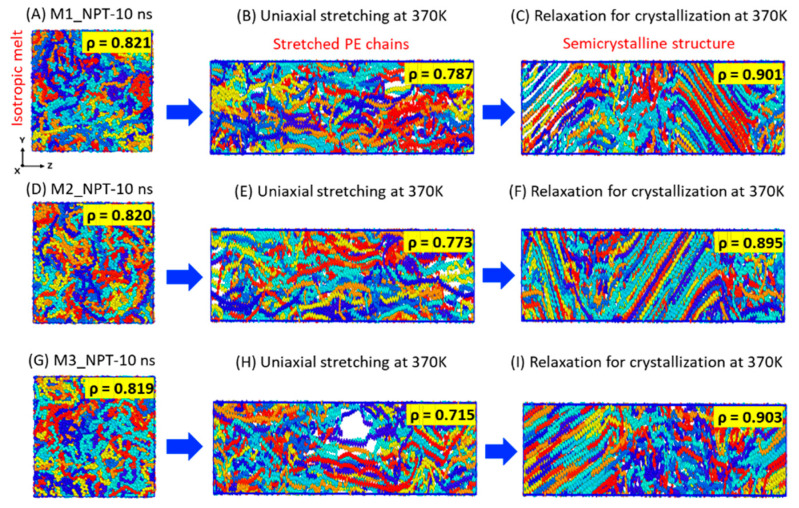
Structural characteristics of semicrystalline model preparation, starting from well-equilibrated melt models (**A**,**D**,**G**), followed by stretching models up to 300% (**B**,**E**,**H**), and concluding with relaxed models showing stable lamellae formation (**C**,**F**,**I**).

Figure 3 illustrates the structural changes during relaxation at 370 K for 10 ns, followed by stretching up to 300%, and, finally, the alignment of the chains due to 100 ns relaxation at the same temperature. A quantitative measurement of the formation of each state at 370 K is indicated by the density data in Figure 3, which are ~0.820 g·cm^−3^ for the isotropically melted model, ~0.775 g·cm^−3^ for the stretched chains, and ~0.90 g·cm^−3^ for the final semicrystalline structure after 100 ns of relaxation. Moreover, the stretched chains in Figure 3B,E are well aligned along the stretching direction, in contrast to those in Figure 3H, which formed a structural void. We ensured no breakage of the chains, and the model in Figure 3H finally stabilized into a semicrystalline structure with the desired densities (~0.90 g·cm^−3^), comparable to similar models obtained without any voids. All the semicrystalline models in the relaxation process are well stabilized within a time length of 100 ns, suggesting that 300% elongation for crystallization in SIC is sufficient to obtain a suitable semicrystalline model with moderate densities and a balanced distribution of amorphous and crystalline regions, even under the high chain mobility observed at 370 K.

#### 3.2.2. Structure and Chain Conformation

**Figure 4:**
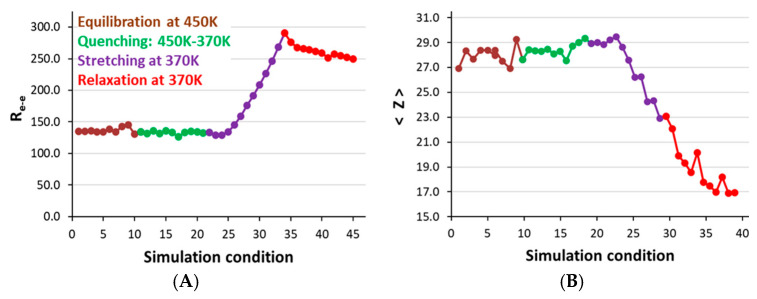
(**A**) The average chain end-to-end distances in Å under different simulation conditions. (**B**) The average number of entanglements per chain (<*Z*>) across all three models during equilibration, quenching, and 300% stretching at T = 370 K and P = 1 atm. All parameters represent the averages of three independent calculations.

MD simulations can capture chain dynamics during crystallization with the rapid cooling method. This is a crucial aspect of this tool. The analysis of HDPE microstructure throughout all simulation steps is reported in Figure 4 by computing the chain end-to-end distance ($R_{ee}$ and the average number of entanglements per chain, <*Z*>). These two parameters are calculated using the *Z*1+ code by Kröger et al. [51,52]. As shown in Figure 4, the $R_{ee}\;$ and <*Z*> values are stabilized and remain consistent during the equilibration at 450 K, the quenching stages from 450 K to 370 K, and even the subsequent relaxation state at 370 K for 10 ns, before the stretching. In fact, due to sufficient relaxation at 370 K, the <*Z*> value was slightly increased due to the loss of chain mobility and close chain packing.

During the stretching with a high strain rate of 10^8^ s^−1^, the extended chain’s $R_{ee}$ value considerably increased from 134 to 291 Å. At the same time, <*Z*> decreased from 29.0 to 23.0, reflecting the high chain alignment conformation due to stretching and loss of coil chain structure, and each chain is elongated along the deformation axis with high external forces. The$\;R_{ee}$ value after stretching is close to 291 Å, indicating a highly stretchable PE chain conformation. On the other hand, the chain conformation during stretching forms a fibril structure due to the loss of the chain-folded conformation. It has a less entangled chain structure. As these models are relaxed in the subsequent steps, a slight decrease in $R_{ee}$ and a further reduction in <*Z*> were observed, further suggesting that the relaxation process is saturated with the $R_{ee}$ value of ~250 Å. The chain’s further relaxation could not give a chain-folded or globular conformation, as the stretched chains remained highly aligned along the deformation axis. The <*Z*> value reached ~17.0 from 23.0 after relaxation, since the stretched chains aligned and the lamellar conformation thickened, further preventing entanglement formation in the semicrystalline model structure obtained using the SIC method [15,16,55,56,57,58,59].

**Figure 5:**
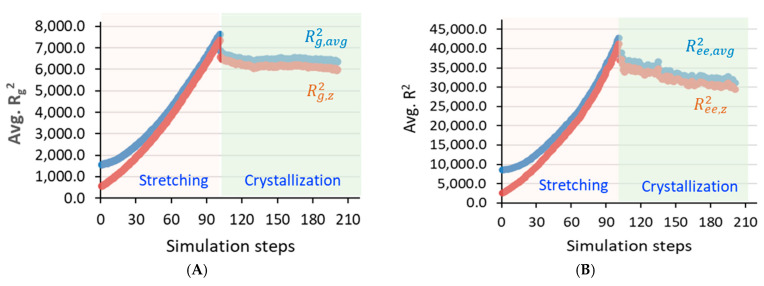
The squares of the average radius of gyration (**A**) and average chain end-to-end distances (**B**) for all models at 370 K.

More specifically, the squares of the total average radius of gyration (Avg. $R_{g}^{2}$), its *Z*-axis component ($R_{g,z}^{2}$), and the average chain end-to-end distances (Avg. $R_{ee}^{2}$) were computed to gain more information on the microstructure characteristics in the SIC process with high chain mobility. The increase in $R_{g}^{2}$ and $R_{g,z}^{2}$ is abrupt upon stretching and reflects the highly stretchable polymer chain conformation observed in fiber materials. Both $R_{g}^{2}$ and $R_{g,z}^{2}$ values in Figure 5 show a maximum elongation of HDPE chains, and upon relaxation, their chain conformation is slightly quenched; the chain elongation conformation is retained in the model and helps in the lamellar thickening process. The elongation occurs only along the *z*-axis, with minimal changes in the other two directions (x and y), suggesting the formation of an anisotropic conformational model, an expected morphology in SIC processes [10].

### 3.3. Degree of Crystallinity ($\chi_{c}$ *in %)*

**Figure 6:**
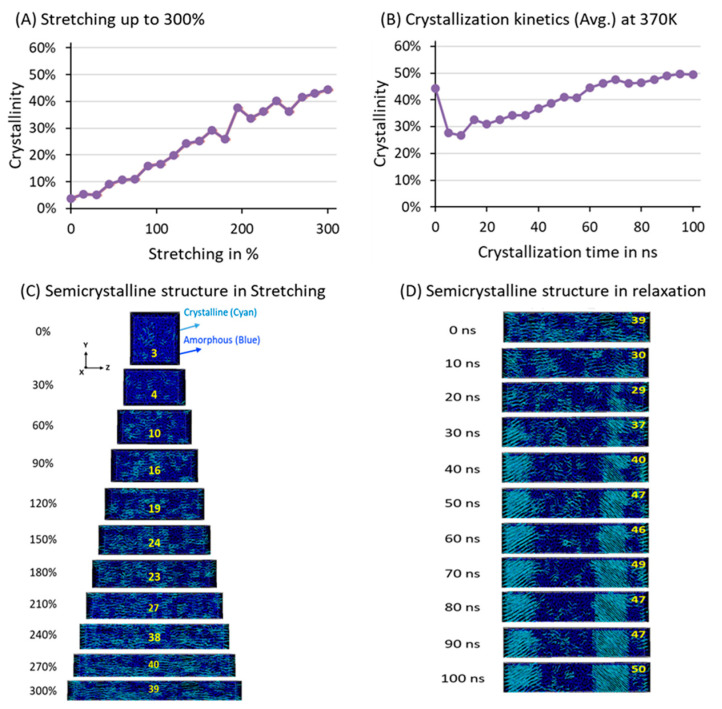
Average degree of crystallinity during the 300% stretching process (**A**) and during relaxation for 100 ns (**B**). Representation of the lamellar orientation in the 300% stretching model (**C**) and its relaxed-state models for 100 ns (**D**). The navy blue and cyan colors illustrate the amorphous and crystalline orientations.

Figure 6 reports the degree of crystallinity in the phase transformation during the stretching (Figure 6A) and relaxation (Figure 6B) processes at 370 K. At the beginning of the stretching, $\chi_{c}$ is ~3%, indicating that the lamellar chain formation is negligible. Figure 6A illustrates that $\chi_{c}\;$ increases to 39% and further rises to 50% after 100 ns relaxation due to the thickening of the lamellar stems upon relaxation and loss of elongational stress. The $\chi_{c}$ in the SIC process gradually increased from the beginning of the stretching deformation, whereas the relaxation process showed a slow growth, which indicated that the HDPE chains in the stretching were aligned as the strain increased and changed into a highly aligned conformation along the deformation axis. The stretched model was less entangled, and due to the 300% deformation, a high stress was developed, which was suddenly removed as the relaxation process proceeded. Later, the elongated PE chains slowly thickened, which resulted in the strengthening of the lamellae stem and a simultaneous increase in $\chi_{c}$ to the final value of 50%. Figure 6C,D represent the crystal formation during stretching and lamellae thickening, respectively. The lamellar orientation in the relaxation process has been tilted to the deformation *Z*-axis.

### 3.4. Lamellar Orientation Analysis

**Figure 7:**
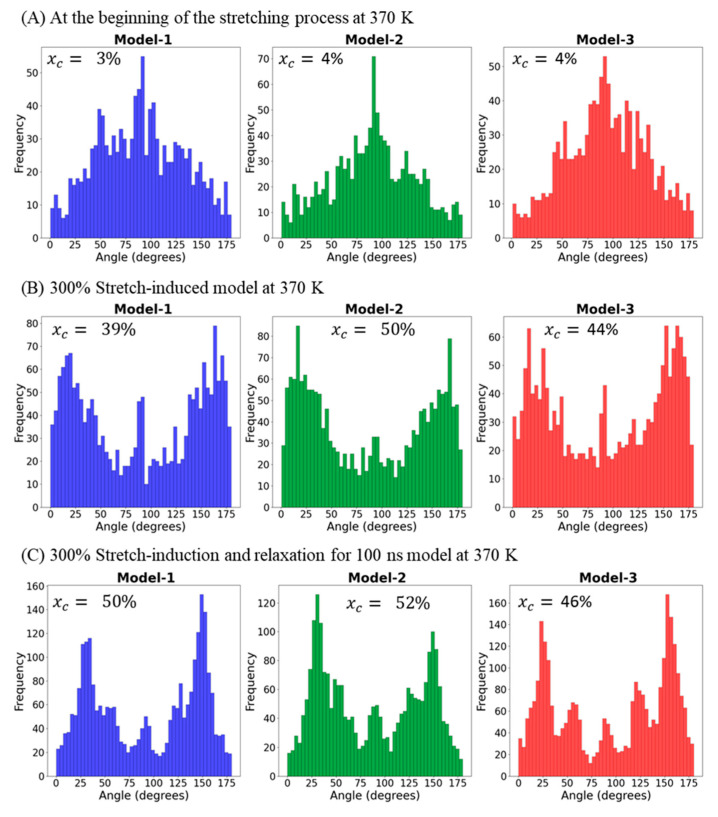
Lamellar tilt angle (*θ*) along the deformation axis (Z) for all three models: the 370 K relaxed model (**A**), the 300%-stretched model at 370 K (**B**), and the 100 ns relaxed model at 370 K (**C**).

The distribution of lamellar orientation in all three semicrystalline models is calculated using self-developed Python code under three simulation conditions: lamellar orientation along the deformation axis (*Z*-axis) is reported for the 10 ns equilibrated models at 370 K (Figure 7A), the 300% stretch-induced model at 370 K (Figure 7B), and models after performing 100 ns relaxation MD at 370 K (Figure 7C). The final MD simulation snapshot geometry is considered for lamellar orientation analysis by estimating the tilt angle (θ) between the lamellar stem and the stretching *Z*-axis.

The code initially distinguishes between the densely packed crystalline regions and the mixed orientation in the simulation box. Generally, the crystalline regions exhibit a high local density distribution and ordered packing; crystalline atoms are classified based on having 10 or more neighboring atoms within a 5 Å radius. The vector difference between consecutive crystalline atoms is computed. For the semicrystalline models, the code considers angles between 0° and 180° to account for the preferred lamellar orientation. The orientation angle is computed using the dot product, as shown in Equation (9):

(9) $$\cos\theta =\frac{\vec{\vartheta }.\vec{z}}{\left\|\vec{\vartheta }\right\|\left\|\vec{z}\right\|}$$

where $\vec{\vartheta }$ is the lamellar vector; $\vec{z}$ is the deformation axis vector. The forward lamellar tilt is *θ* degrees relative to the *z*-axis. The backward tilt is calculated as 180°–*θ* degrees.

Figure 7 illustrates the preferential orientation of the HDPE chains in sections A to C. The PE chain orientations in Figure 7A–C exhibit characteristic bar diagrams. The distinct variations observed in these three images illustrate molecular insights into the chains as they undergo deformations and relaxations. Specifically, Figure 7A illustrates a Gaussian curve for the equilibrated model at 370 K before applying the stretching deformation. This can be attributed to the random orientation of the atoms to the deformation axis, supporting the model’s total density (ρ = ~0.82 g·cm^−3^) and degree of crystallinity (χ_c_ 3–4%). The density of HDPE at room temperature (R.T.) is known to be 0.85 g·cm^−3^, and with a slight increase in temperature to 370 K, it decreased to 0.820 g·cm^−3^, indicating greater chain flexibility. The increased crystallinity serves as a clear indicator of the elongation of lamellar stems along the deformation axis, while the bimodal lamellar distribution in Figure 7B,C shows that the lamellae orientation is symmetrically tilted in the relaxed model at 370 K after stretching at the same temperature, i.e., 370 K. The presence of bimodal peaks confirms the characteristics of true semicrystalline materials, which naturally consist of alternating lamellae and amorphous regions. The tilt angles of the stacked lamellae orientation range between 20–35° and 130–145°, resembling the tilting angles reported in experimental (~35°) and computational studies (20–24°) on HDPE regarding the lamellae orientation to the deformation *z*-axis [3,27]. This bimodal lamellar distribution is thought to relate to the intrinsic chain folding behavior of the material.

### 3.5. Mechanical Properties

**Figure 8:**
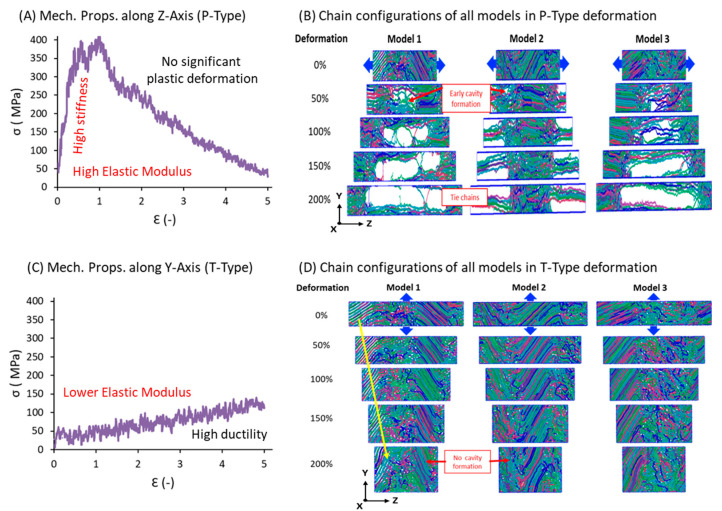
S-S curves and structural deformation during uniaxial elongation along the *Z*-axis for lamellar orientations: P-type elongation (**A**,**B**) and T-type elongation (**C**,**D**).

Tensile tests were performed along and parallel to the deformation axis to account for the anisotropic morphological distribution in the semicrystalline models. Uniaxial elongation in the tensile tests along the *z*-axis, where chains are highly stretched and relaxed in the SIC method, is known as parallel deformation (P-type). In contrast, deformation perpendicular to the *z*-axis, where elongation occurs in the *x-* and *y*-axis directions, is known as transverse-type (T-type) deformation. The results of the above two deformation types for the three samples are reported as stress–strain curves (SSCs) in Figure 8A,C. The corresponding structural evaluations during deformation are shown in Figure 8B,D.

A semicrystalline model with a fibril structure is shown in the P-type deformation SS curve. P-type elongation along the *z*-axis shows a high elastic modulus of ~350–400 MPa, which is quite high for PE, indicating the presence of a fibril structure where lamellar stems are highly aligned along the deformation direction and covalent sigma bonds are resisting the elongation forces. The resistance by the direct covalent bond forces shows a much higher elastic regime and higher Young’s modulus, accompanied by a breakage point approximately at a strain of 1.5, a typical cavitation molecular mechanism observed in semicrystalline materials. The true SSC is mimicked in the tensile test of the obtained model, supporting the high fibril structure formation in this process, indicating that higher-temperature stretching has not affected the inner structure of the semicrystalline material, and that the 370 K and 1 atm condition can also give a strong fibril HDPE model. Additionally, as we have seen, void formation occurred in the model-3 case when it was stretched up to 300% and formed a stable semicrystalline model upon relaxation. Structural analysis in Figure 8B evidences that the SS curve has characteristic semicrystalline features and that 100 ns of relaxation at 370 K is sufficient to form a stable semicrystalline model, supporting 370 K and 1 atm as suitable conditions for model formation.

Out of curiosity and to explore more structural characteristics of the model, we applied deformation forces perpendicular (transverse direction) to the deformation axis along the *x*/*y*-axes. Since the model is highly aligned along the *z*-axis, the remaining axes appeared to be similar, and the results are shown in Figure 8C,D for T-type deformation. Unlike the P-type SSC, the T-type SS curve represents amorphous model deformation, where the Young’s modulus is lower and the elastic regime is less stiff [10]. Further, this curve displays an elongated, durable behavior due to weak van der Waals forces bearing the extension load applied in the transverse direction. The resulting elastic regime is 50 MPa, which is eight times lower than that observed in the P-type elongational tensile test. The structural characteristics also reflect that the deformation did not show any voids or cavitation in the P-type deformation, even up to 500% strain, indicating the absence of lamellar orientation along the *x*/*y*-axis directions. This demonstrates the anisotropic distribution or orientation of lamellar stems, which is a characteristic property of the model obtained from SIC simulations of semicrystalline models.

## 4. Conclusions

This study used MD simulations to reveal the SIC behavior of 10C_1000_ HDPE models at 370 K. The stretching temperature (370 K) just below the melting point temperature of PE was chosen to capture insightful details of crystallization in HDPE, where chain mobility is higher than the reported crystallization temperature (~350 K) for similar SIC models. The selected model sizes are slightly smaller, but qualitative information was obtained under each simulation condition, including equilibration, stretching, and relaxation during semicrystalline structure formation. This investigation revealed that crystallization at 370 K is a little slower than in lower-temperature crystallization models. This could be attributed to the inhibition of fast chain alignment due to high chain mobility. Although 100 ns relaxation simulations were performed immediately after 300% stretching, the degree of crystallization was lower than that reported in earlier models at 350 K [1,10,30,55]. The degree of crystallization was ~50% ± 5%, which reflects that the amorphous and crystalline regions co-existed in nearly equal proportions, which can be anticipated as a way to prepare a semicrystalline model. The morphology was anisotropic, and lamellar stems were highly stretched and elongated along the deformation *z*-axis. However, chain tilt angle analysis revealed that the stretched chains along the *z*-axis were slightly tilted between 20 and 35°, which corroborated the experimental value of < 35° [27] and simulated value of 25° [2,3]. Despite the evenly distributed morphology of highly elongated PE chains, the elastic region exhibits a Young’s modulus of ~350 MPa when HDPE semicrystalline chains are elongated along the deformation axis (P-type). At the same time, a breakage point is also observed, followed by void formation after 120–150% deformation. On the other hand, T-type deformation displays an elastomeric SSC characteristic, which is due to the elongation along non-covalent bonds, resulting in a low elastic regime, and the deformation is stretchable, which does not reflect the typical behavior of semicrystalline materials in tensile tests. [10] The P-type SS curve provides the fiber with material characteristics [4]. This is also consistent with models studied at 350 K, confirming the reliability of model preparation at 370 K, which could be an optimal way to study the crystallization characteristics of HDPE fiber materials.

## Figures and Tables

**Table 1:** The parameters of the Trappe-UA forcefield, along with their real units for both bonded and non-bonded terms, are displayed. All parameter units are presented in the real units used by the LAMMPS simulation code.

Bonded	
Functional form	Parameters
Bond potential	
K_b_ (kcal/mol)	260.00013
R_0_ (Å)	1.54
Angle potential	
K_θ_ (kcal/mol/rad^2^) for CH_2_-CH_2_ and CH_3_-CH_3_	62.1002
*θ* (Degree) for CH_2_-CH_2_ and CH_3_-CH_3_	114.0
Dihedral potential	
Zeroth-order term: *C*_0_ (kcal/mol)	2.00702
First-order term: *C*_1_ (kcal/mol)	−4.01203
Second-order term: *C*_2_ (kcal/mol)	0.27102
Third-order term: *C*_3_ (kcal/mol)	6.29006
**Non-bonded**	
*ε* (kcal/mol) for CH_2_-CH_2_	0.09141
*ε* (kcal/mol) for CH_3_-CH_3_	0.19475
*σ* (Å) for CH_2_-CH_2_	3.95
*σ* (Å) for CH_3_-CH_3_	3.75

## Data Availability

The data supporting this study’s findings are available upon request from the corresponding author.
